# A novel TCGA-validated programmed cell-death-related signature of ovarian cancer

**DOI:** 10.1186/s12885-024-12245-2

**Published:** 2024-04-23

**Authors:** Xintong Cai, Jie Lin, Li Liu, Jianfeng Zheng, Qinying Liu, Liyan Ji, Yang Sun

**Affiliations:** 1 Department of Gynecology, Clinical Oncology School of Fujian Medical University, Fujian Cancer Hospital, Fuzhou, Fujian Province China; 2 Fujian Provincial Key Laboratory of Tumor Biotherapy, Clinical Oncology School of Fujian Medical University, Fujian Cancer Hospital, Fuzhou, Fujian Province China; 3 Geneplus-Beijing Institute, Beijing, China

**Keywords:** Programmed cell-death, Ovarian cancer, Risk model, TCGA, Drug sensitivity, Immunotherapy

## Abstract

**Background:**

Ovarian cancer (OC) is a gynecological malignancy tumor with high recurrence and mortality rates. Programmed cell death (PCD) is an essential regulator in cancer metabolism, whose functions are still unknown in OC. Therefore, it is vital to determine the prognostic value and therapy response of PCD-related genes in OC.

**Methods:**

By mining The Cancer Genome Atlas (TCGA), Genotype-Tissue Expression (GTEx) and Genecards databases, we constructed a prognostic PCD-related genes model and performed Kaplan-Meier (K-M) analysis and Receiver Operating Characteristic (ROC) curve for its predictive ability. A nomogram was created via Cox regression. We validated our model in train and test sets. Quantitative real-time PCR (qRT-PCR) was applied to identify the expression of our model genes. Finally, we analyzed functional analysis, immune infiltration, genomic mutation, tumor mutational burden (TMB) and drug sensitivity of patients in low- and high-risk group based on median scores.

**Results:**

A ten-PCD-related gene signature including protein phosphatase 1 regulatory subunit 15 A (PPP1R15A), 8-oxoguanine-DNA glycosylase (OGG1), HECT and RLD domain containing E3 ubiquitin protein ligase family member 1 (HERC1), Caspase-2.(CASP2), Caspase activity and apoptosis inhibitor 1(CAAP1), RB transcriptional corepressor 1(RB1), Z-DNA binding protein 1 (ZBP1), CD3-epsilon (CD3E), Clathrin heavy chain like 1(CLTCL1), and CCAAT/enhancer-binding protein beta (CEBPB) was constructed. Risk score performed well with good area under curve (AUC) (AUC_3 − year_ =0.728, AUC_5 − year_ = 0.730). The nomogram based on risk score has good performance in predicting the prognosis of OC patients (AUC_1 − year_ =0.781, AUC_3 − year_ =0.759, AUC_5 − year_ = 0.670). Kyoto encyclopedia of genes and genomes (KEGG) analysis showed that the erythroblastic leukemia viral oncogene homolog (ERBB) signaling pathway and focal adhesion were enriched in the high-risk group. Meanwhile, patients with high-risk scores had worse OS. In addition, patients with low-risk scores had higher immune-infiltrating cells and enhanced expression of checkpoints, programmed cell death 1 ligand 1 (PD-L1), indoleamine 2,3-dioxygenase 1 (IDO-1) and lymphocyte activation gene-3 (LAG3), and were more sensitive to A.443,654, GDC.0449, paclitaxel, gefitinib and cisplatin. Finally, qRT-PCR confirmed RB1, CAAP1, ZBP1, CEBPB and CLTCL1 over-expressed, while PPP1R15A, OGG1, CASP2, CD3E and HERC1 under-expressed in OC cell lines.

**Conclusion:**

Our model could precisely predict the prognosis, immune status and drug sensitivity of OC patients.

**Supplementary Information:**

The online version contains supplementary material available at 10.1186/s12885-024-12245-2.

## Introduction

Ovarian cancer (OC) is the third most common gynecologic malignancy worldwide but accounts for the highest mortality [1]. The common symptoms are often deceptive, such as bloating, early satiety, and discomfort in the stomach [2]. Patients are always diagnosed in the advanced stages. Although OC has many subtypes, such as epithelial carcinoma, germ cell tumor, sex cord-stromal tumor, and Krukenberg’s tumor [3], the treatment is almost consistent. According to NCCN guidelines, the standard treatment is entirely cytoreduction surgical, followed by adjuvant chemotherapy. Maintenance therapies with poly ADP-ribose polymerase (PARP) inhibitors and bevacizumab are increasingly widely used [4]. Recent treatments combined with immune checkpoint blockade, PARP inhibition, chemotherapy, and antiangiogenic drugs have been widely encouraged in clinical trials for OC patients with advanced and metastatic stages. Still, the outcomes are inferior [5]. To date, accumulating evidence suggests that the high recurrence rate is thought to be due to remaining drug-resistant cells and cancer stem cells (CSC) [6]. In addition, OC cells may undergo an immunoediting process that orchestrates the interaction between the infiltrating immune cells and ovarian stromal microenvironment to promote tumor progression [7]. Despite the considerable advancement in OC treatment, there was no noticeable improvement in recurrence and survival rates [8]. Therefore, finding novel biomarkers to aid in the prognosis prediction and treatment of OC is increasingly essential.

**Fig. 1:**
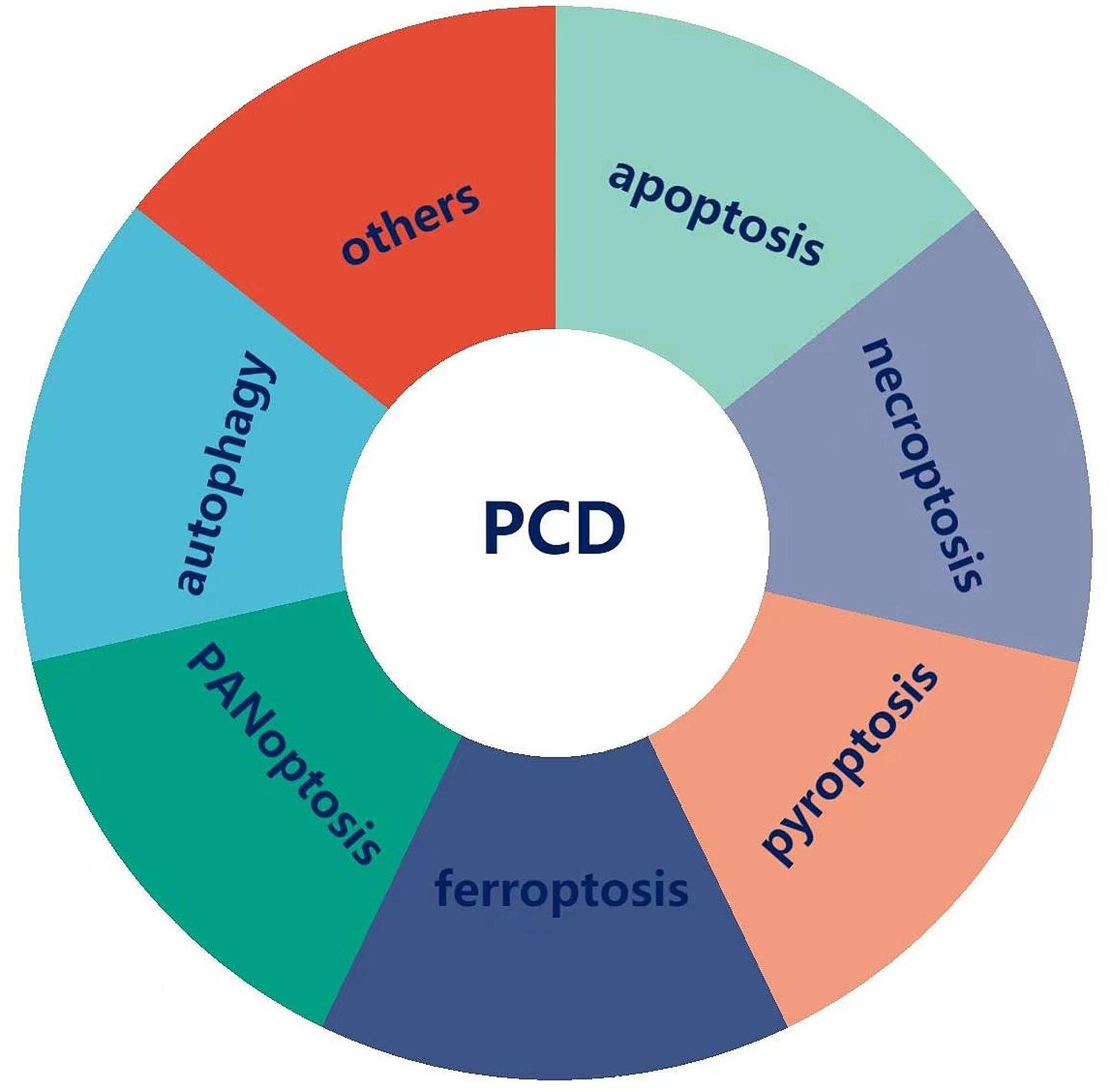
The main physiological ways included in programmed cell death (PCD)

Cell death is a fundamental physiological process in all living organisms, from embryonic development, organ maintenance, tumorigenesis, and immune responses [9]. Cell death occurs in two significant ways—accident cell death (ACD) and programmed cell death (PCD). ACD is a biological process that happens to lose control. However, PCD commanded acceptable regulations and interplay with various mechanisms. PCD consists of apoptosis, necroptosis, pyroptosis, ferroptosis, PANoptosis, and autophagy (Fig. 1) [10]. PCD is crucial in modulating the immunosuppressive tumor microenvironment (TME) and determining clinical outcomes of treatments [11]. Many scholars have confirmed apoptosis and autophagy can collaborate to reverse chemoresistance, reduce metastasis, and improve prognosis in OC patients [12–14]. Tan C. et al. also found inhibition of pyroptosis promoted OC tumor progression by regulating the ASK1/JNK signaling pathway [15]. These studies provide new insights into crucial player roles of certain forms of PCD in prognosis prediction and treatment plan selection. Hence, investigating PCD-related genes may help clinicians predict survival outcomes and make personalized treatment plans in OC patients.

Our study aims to construct a PCD-related gene signature using The Cancer Genome Atlas (TCGA) database. We assessed its value in prognosis prediction, potential target drugs, and immune responses to help clinicians predict the prognosis and make personalized treatment plans for OC patients.

**Fig. 2:**
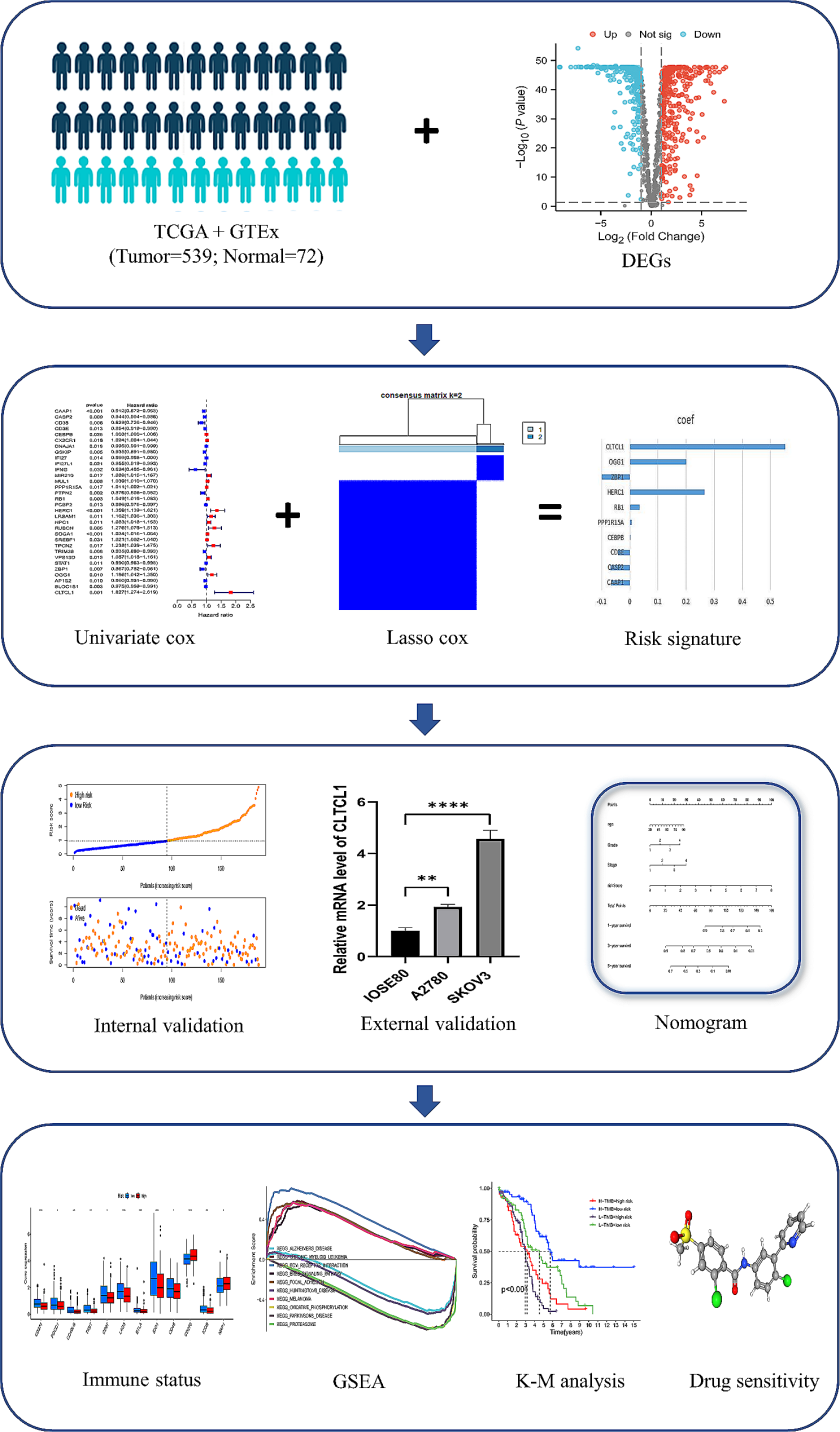
Flowchart for comprehensive analysis of PCD patterns in OC

## Materials and methods

### Data source

Clinical information and RNA-seq profiles of OC patients were downloaded from the TCGA database (https://www.cancer.gov/, accession number: phs000178). The clinical features are detailed in supplementary Table S1. Information about normal ovary tissue was obtained from GTEx (https://xenabrowser.net/datapages/?cohort=GTEX&removeHub=143 https%3 A%2 F%2Fxena.treehouse.gi.ucsc.edu%3A443). All data were downloaded and collated using Perl language (v5.30.0) and R language (v4.1.2). PCD genes were retrieved from the GeneCards database (https://www.genecards.org/Search/Keyword?queryString=programmed%20cell%20death). The symbol of mRNAs was annotated using the ensembles human genome browser GRCh38. p13 (http://asia.ensembl.org/index.html).

### Differentially-expressed genes of programmed cell-death in ovarian cancer

We conducted a differentially-expressed analysis of PCD genes by comparing 72 normal and 539 tumor tissue with R package “limma”. *P* < 0.05 and|log2FC| > 1. Then, we performed Gene Ontology (GO) and Kyoto Encyclopedia of Genes and Genomes (KEGG) analysis to search for potential biological functions and mechanisms. Continually, we used the STRING database to analyze protein-protein interaction (PPI). At last, we used Cytoscape to visualize the relationship of the top ten hub genes.

### Cluster analysis

Using Univariate Cox regression analysis, we screened out DEGs of PCD related to the prognosis of OC patients. We also identified PCD-related molecule subtypes (two clusters) using the “ConsensusClusterPlus” package (maxK = 9), which was established on the parameters of “clusterAlg” selected as “km”, and “distance” selected as “euclidean”. Then we compared the prognosis between the two clusters. Further, Kaplan-Meier (K-M) analysis was used to compare the prognosis between the two clusters. Continually, the heatmap displayed the correlation between clusters and clinical parameters, such as (grade, age, fustat, and stage), which was analyzed by chi-square test. Lastly, we analyzed the relationship between clusters and immune conditions.

### Predictive model construction

We obtained ten PCD-related genes using multivariate Cox regression and LASSO analysis and constructed a prognostic model. The coefficient of the selected genes was displayed by Graphpad software. Then, we evaluated the prognostic value using K-M analysis and the ROC curve. We also validated the reliability and stability of our model by randomly separating OC patients (entire set *n* = 378) into a train set (*n* = 190) and a test set (*n* = 188) based on R package “caret”. Furthermore, we estimated whether our signature was an independent risk factor by univariate and multivariate Cox analysis and built a nomogram using “rms” R package. Based on clinicopathological parameters, calibration plots were used to compare the consistency between predicted probabilities of 1-, 3- and 5-year survival.

### Functional analysis and immune landscape analysis

Patients were divided into high and low-risk groups based on the median risk score. We conducted GSEA to analyze the potential pathways enriched in GO and KEGG gene sets [16]. The screening conditions were|normalized enrichment score (NES)| > 1, nominal (NOM) *p*-value < 0.05 and FDR q-value < 0.25. The activity of TME, immune infiltrating cells, immune function, and immune checkpoints were analyzed. In two risk groups, ESTIMATE was used to calculate the immune score, stromal score, estimate score, and tumor purity. The immune infiltrating cells were calculated by single sample GSEA (ssGSEA) and TIMER database via the “GSVA” R package. Immune checkpoints were also compared in two groups. Differential functions were analyzed using the Wilcoxon rank-sum test between two groups.

### Gene mutation analysis

Using the “maftools” package, we conducted gene mutation based on somatic mutation data. We then calculated each OC patient’s TMB and compared it between two risk groups. Furthermore, we conducted a survival analysis based on different TMB groups and subgroups. We also displayed somatic mutations of the selected genes using the cBioPortal database.

### Chemotherapy sensitivity and small molecule drugs

The Genomics of Drug Sensitivity in Cancer (GDSC) database (www.cancerRxgene.org) is the largest public resource for information on drug sensitivity in cancer cells and molecular markers of drug response [17]. The half maximal inhibitory concentration (IC_50_) is commonly used to measure drug effectiveness. The R package pRRophetic (v.0.5, https://osf.io/dwzce/?) was used to predict drug sensitivity by scoring every sample and transferring it to IC_50_ value by calculation, which was described in previous studies [18, 19]. Then, we used the Wilcoxon test to compare scores of certain drugs in high and low-risk groups.

### Quantitative real-time polymerase chain reaction

Human ovary cell line IOSE80 was purchased from Zhejiang Meisen Cell Technology Co., Ltd. Ovarian cancer cell lines (A2780 and SKOV3) were donated by Fujian Provincial Key Laboratory of Tumor Biotherapy. They were cultured under 37℃, 5% CO_2_. The total RNA of three cell lines was refined by instructions of the RNA extraction kit (Promega, LS1040). cDNAs were composed by reverse transcription. We used the SYBR Green Maste20r kit (Roche) for Q-PCR. GAPDH normalized the mRNA expression level of these ten genes. The sequences of primers are listed in supplementary Table 2. GraphPad Prism (v8.0.2) and one-way ANOVA were used for statistical calculation.

### Statistical analysis

All data were downloaded and collated using Perl language (v5.30.0) and R language (v4.1.2). *P* < 0.05 indicated a statistically significant difference. The survival outcomes were compared using K-M analysis. Univariate and multivariate Cox analyses were used to anchor the independent risk factors. GraphPad Prism (V.8.0.2) and one-way ANOVA were used for PCR analysis.

## Results

### Validation of the differentially-expressed genes

**Fig. 3:**
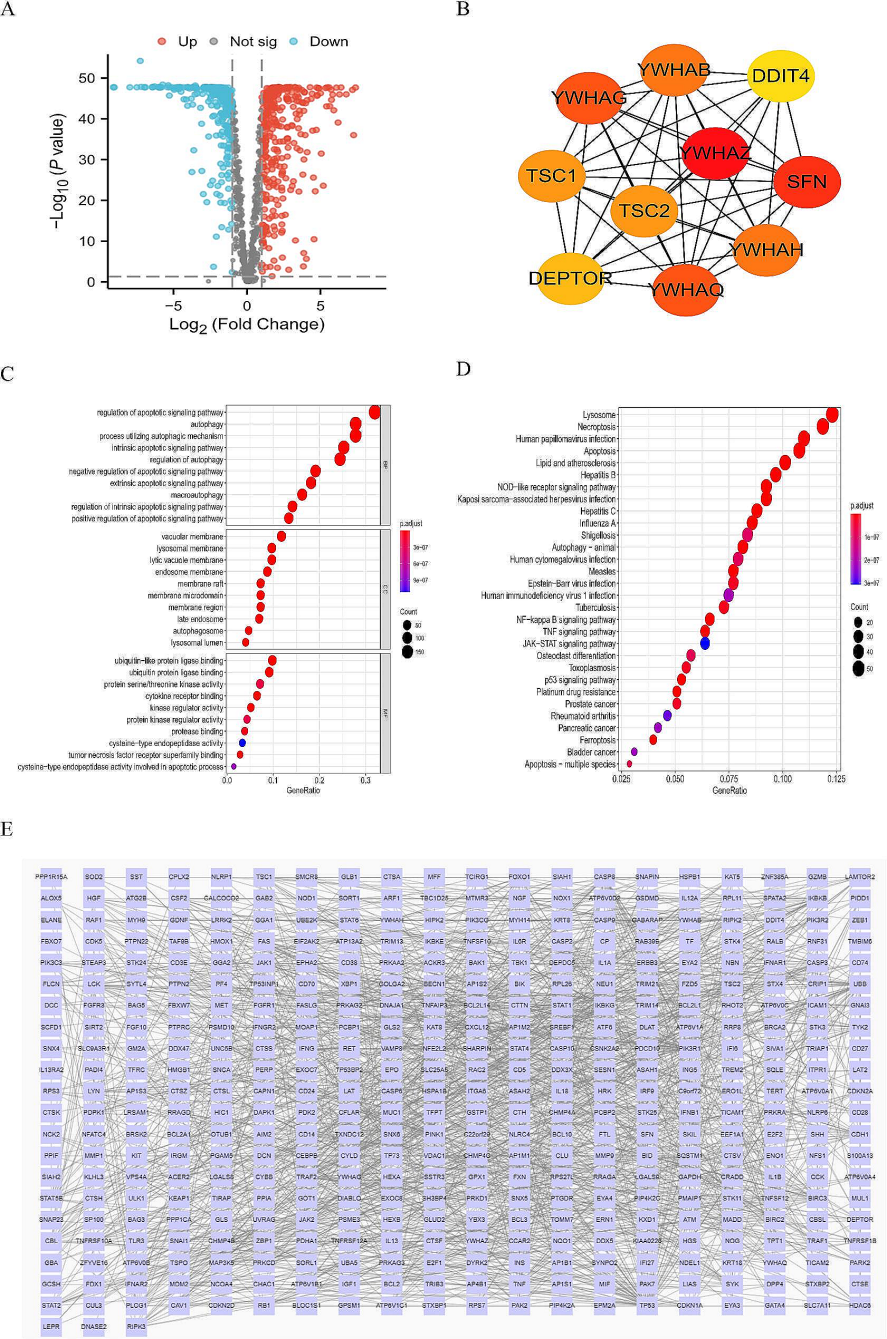
(**A**) Volcano plot of 589 up-regulated and 537 down-regulated DEGs in OC (FDR < 0.05 and|logFC| > 1). (**B**) The interaction relationships between the top ten selected genes. (**C**) GO analysis. (**D**) KEGG analysis. (**E**) PPI network according to the STRING database

The gene expression data of 537 OC patients were downloaded from the TCGA database. After filtering patients with incomplete prognostic information, 378 OC patients were finally selected for our study. Data of 72 normal patients were downloaded from the GTEx database. Also,1254 PCD-related genes were obtained from the Genecards database. The flow diagram is shown in Fig. 2. We finally got 628 DEGs of PCD (345 up-regulated and 283 down-regulated) (*P* < 0.05,|log2FC|>1) (Fig. 3A). The interaction relationships of these top ten hub genes are presented in Fig. 3B. Then, we performed KEGG and GO enrichment analysis. We found those genes were involved in necroptosis, autophagy, and tumor signaling pathways such as NF-kappa B signaling and JAK/STAT (Fig. 3C, D). The PPI network is displayed by Cytoscape from the STRING database (Fig. 3E).

### Two clusters analysis

**Fig. 4:**
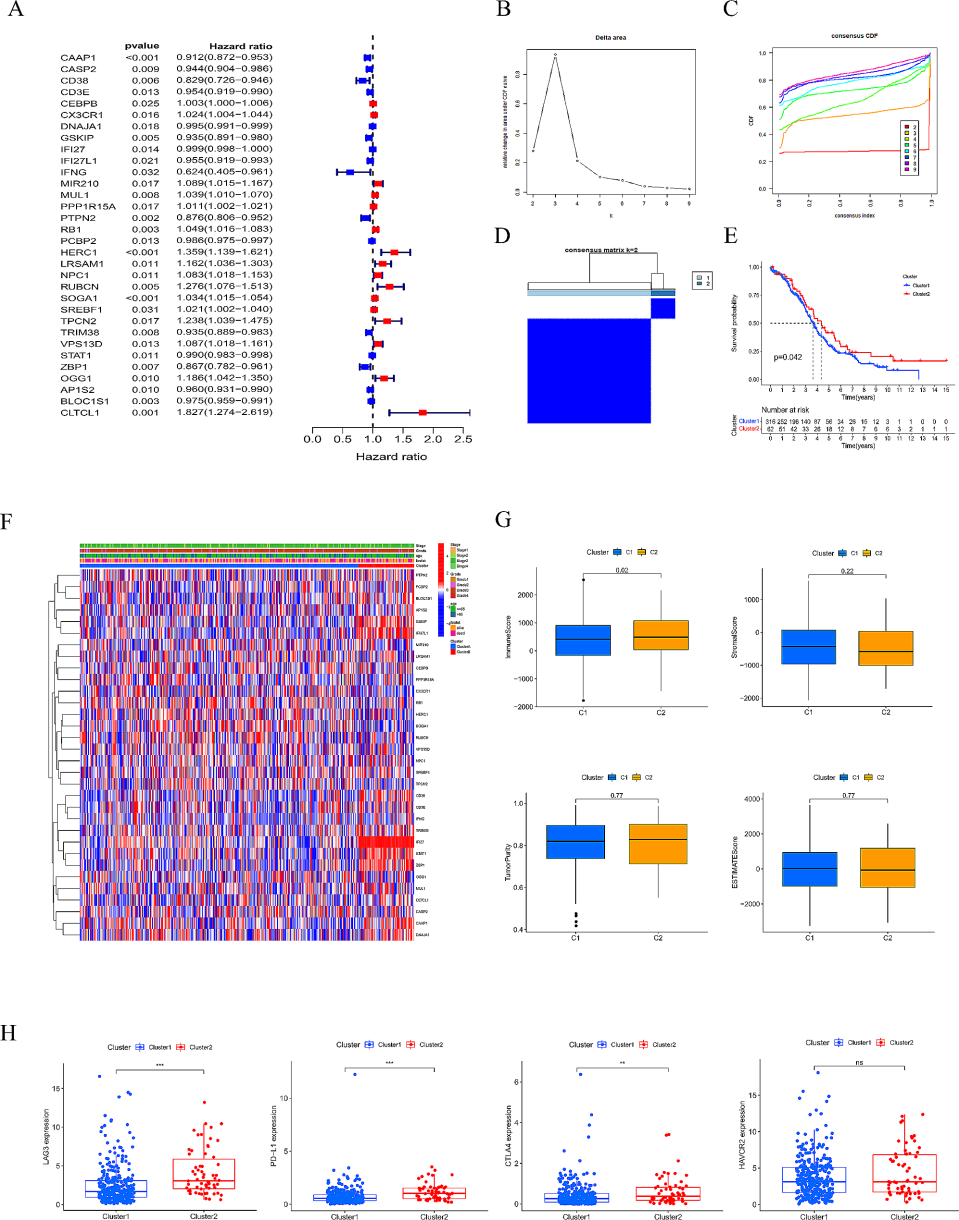
(**A**) Forest plot by univariate Cox analysis. (**B**) Consensus clustering matrix when k = 2. (**C**) Consensus clustering CDF with k valued 2 to 9. (**D**) Relative change in area under CDF curve for k = 2. (**E**) KM curves of the survival in two clusters. (**F**) Heatmap of the ten genes between the two clusters and the correlations of the clusters and clinical parameters. (**G**) Differences in TME of two groups. (**H**) Four common immune inhibitors in two clusters

Using univariate Cox regression analysis, we got 32 prognosis-related genes (Fig. 4A). We divided OC patients into two clusters (cluster 1 and cluster 2) by performing consensus clustering (k = 2) (Fig. 4B, C, D). We demonstrated that patients in cluster 2 sustained more prolonged survival than those in cluster 1 (*P* < 0.05, Fig. 4E). We found higher expression genes such as GSKIP, IFI27L1, CD38, IFI27, STAT1, and Z8P1 in cluster 2 from the clinicopathological parameters heatmap (Fig. 4F). We also searched the relationship between different clusters and immune status. We found cluster 2 shared a higher immune score than cluster 1(*P* < 0.05, Fig. 4G). Immunoinhibitors such as LAG3, PD-L1, and CTLA4 in cluster 2 were higher (Fig. 4H).

### Validation of the predictive signature

**Fig. 5:**
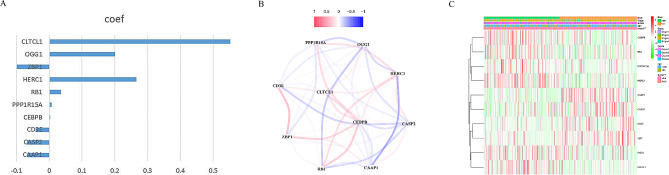
(**A**) Coefficients of the ten genes. (**B**) The correlations between the ten genes. (**C**) Heatmap of 10 genes and clinical features

**Fig. 6:**
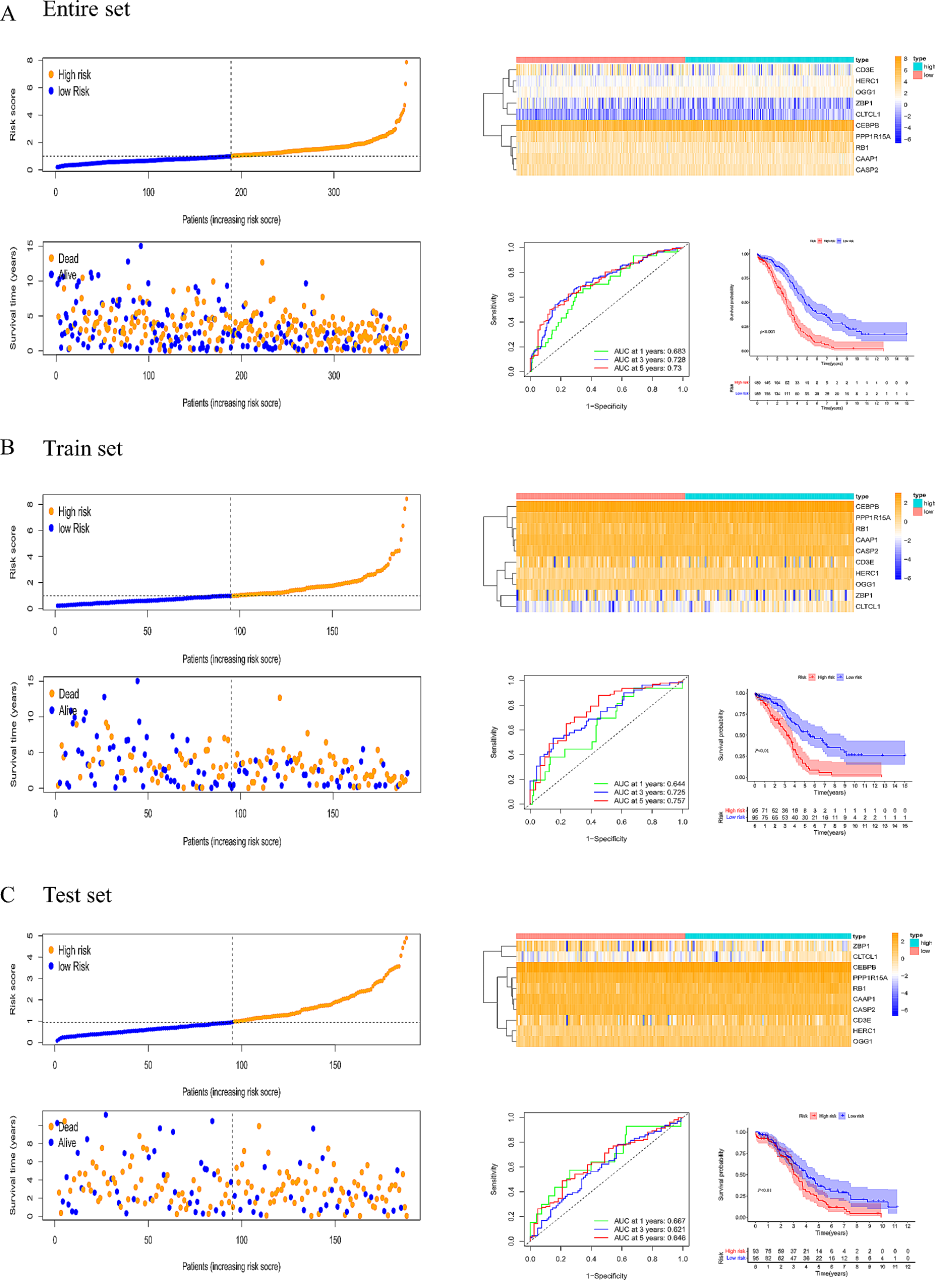
The risk score, heatmap, survival status, clinical outcome, survival analysis and ROC curves in the (**A**) entire set, (**B**) train set and (**C**) test set

**Fig. 7:**
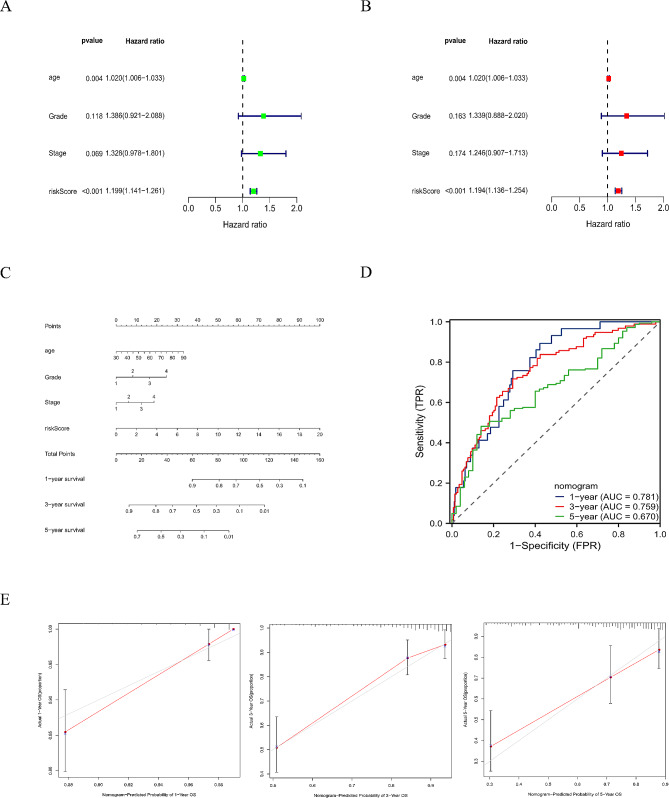
(**A**) Univariate analysis. (**B**) Multivariate Cox analysis. (**C**) Nomogram based on risk score, stage, grade, and age. (**D**) ROC curves of nomogram. (**E**) Calibration plots show the nomogram for predicting OS of 1-, 3- and 5-year

By multivariate Cox analysis, we got ten genes, including PPP1R15A, OGG1, HERC1, CASP2, CAAP1, RB1, ZBP1, CD3E, CLTCL1, and CEBPB (Fig. 5A). The coefficients are shown in Fig. 5B. The risk score for each patient was calculated based on the following formula: Risk Score = coef (Gene_1_)×expression (Gene_1_) + coef (Gene_2_)×expression (Gene_2_) +……+ coef (Gene_n_)×expression (Gene_n_) [20]. We divided patients into high and low-risk groups according to median risk score. The correlations of model genes are displayed in Fig. 5B. We found the expression of CEBPB, RB1, PPP1R15A, HERC1, OGG1, and CLTCL1 was up-regulated in the high-risk group (Fig. 5C). Further, patients in the low-risk group had a better prognosis. ROC curves showed AUC at 1-,3-, and 5- years were 0.683, 0.728, and 0.73, respectively, in the entire set (Fig. 6A) and 0.667, 0.621, and 0.646, respectively, in the test set (Fig. 6C). Similar results were consistent with the train and test sets (Fig. 6B, C). We found that age (*P* < 0.05), clinical stage (*P* < 0.05), and risk score (*P* < 0.01) could be regarded as independent risk factors for OC patients (Fig. 7A). We concluded that our signature was the most valuable factor of the nomogram (Fig. 7B). The calibration plot showed the accuracy of the nomogram for predicting 1-, 3- and 5-year survival times (Fig. 7C). The nomogram’s ROC of 1,3,5-years were 0.781, 0.759, and 0.670, indicating a relatively good prediction ability (Fig. 7D). Calibration plots showed the reliability and stability of our nomogram (Fig. 7E).

### GESA analysis and immune status estimation

**Fig. 8:**
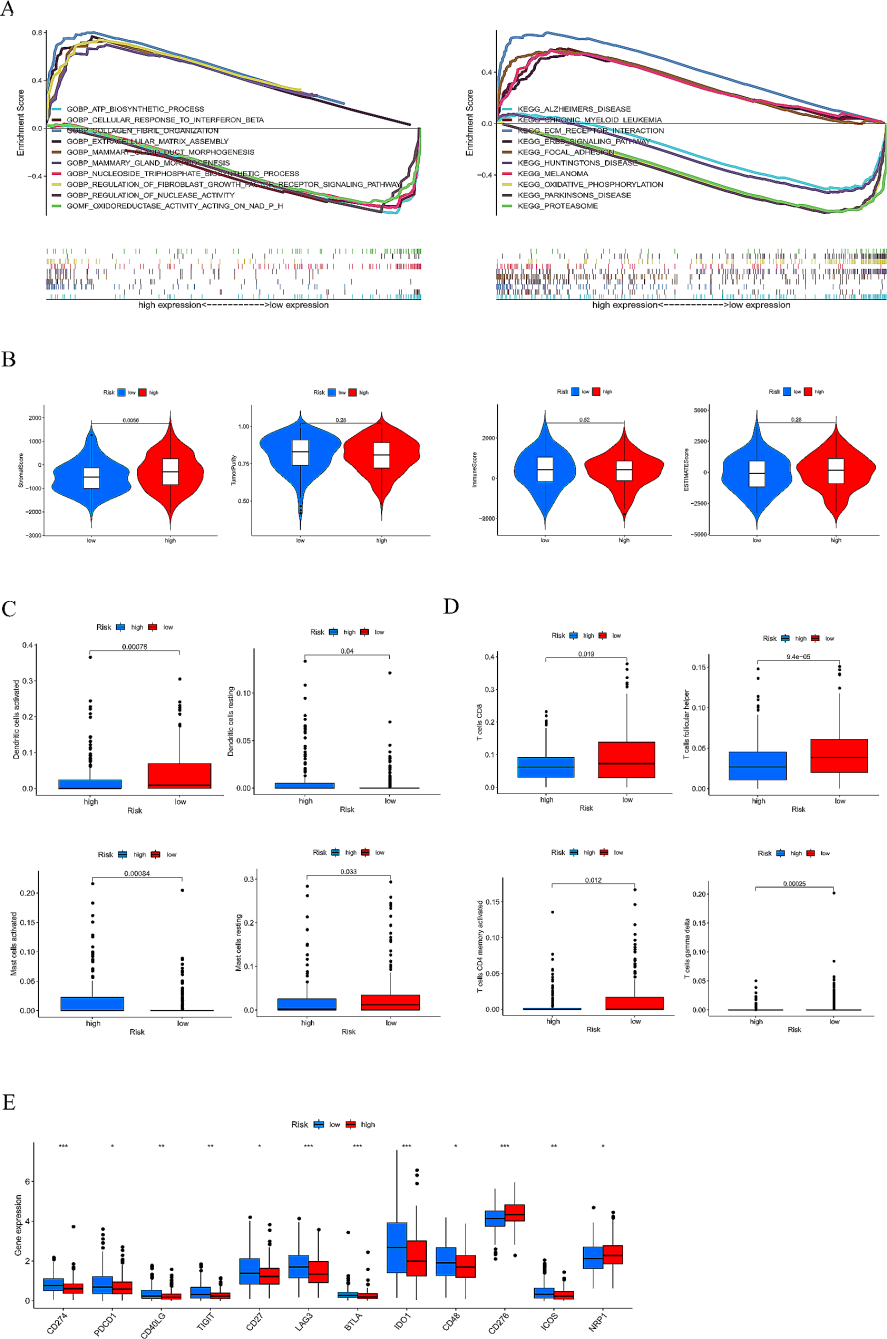
(**A**) Pathways enriched via GO and KEGG analysis in the high-risk group(**B**) TME score in different risk score groups. (**C**,**D**) Immune molecular expression. (**E**) Immune checkpoints. (*P < 0.05; **P < 0.01; ***P < 0.001; ns, not significant)

We found ATP biosynthetic process was the primary physical function in the high-risk group. Also, the ERBB signaling pathway and focal adhesion were highly involved in the high-risk group (Fig. 8A). ERBB signaling pathway is well studied in ovarian cancer. The activation of ERBB could induce EMT, promote cisplatin resistance, and indicate poor prognosis in OC patients [21–23]. The cancer immune environment plays an essential part in tumor progression. Immune infiltrating cells, such as activated dendritic cells, were negatively correlated with low-risk scores (*P* < 0.01), while activated mast cells were conversely (*P* < 0.05) (Fig. 8C). Patients with low-risk scored had higher CD8^+^ T cells (*P* < 0.05), T cells follicular helper (*P* < 0.01), and activated CD4^+^ memory T cells (*P* < 0.05) (Fig. 8D). Subsequently, we found stromal score was higher in the high-risk group (Fig. 8B). The immune checkpoints, such as CD274 and PDCD1, were highly expressed in the low-risk group (Fig. 8E).

### Identification of genomic mutation and tumor mutational burden in the signature

**Fig. 9:**
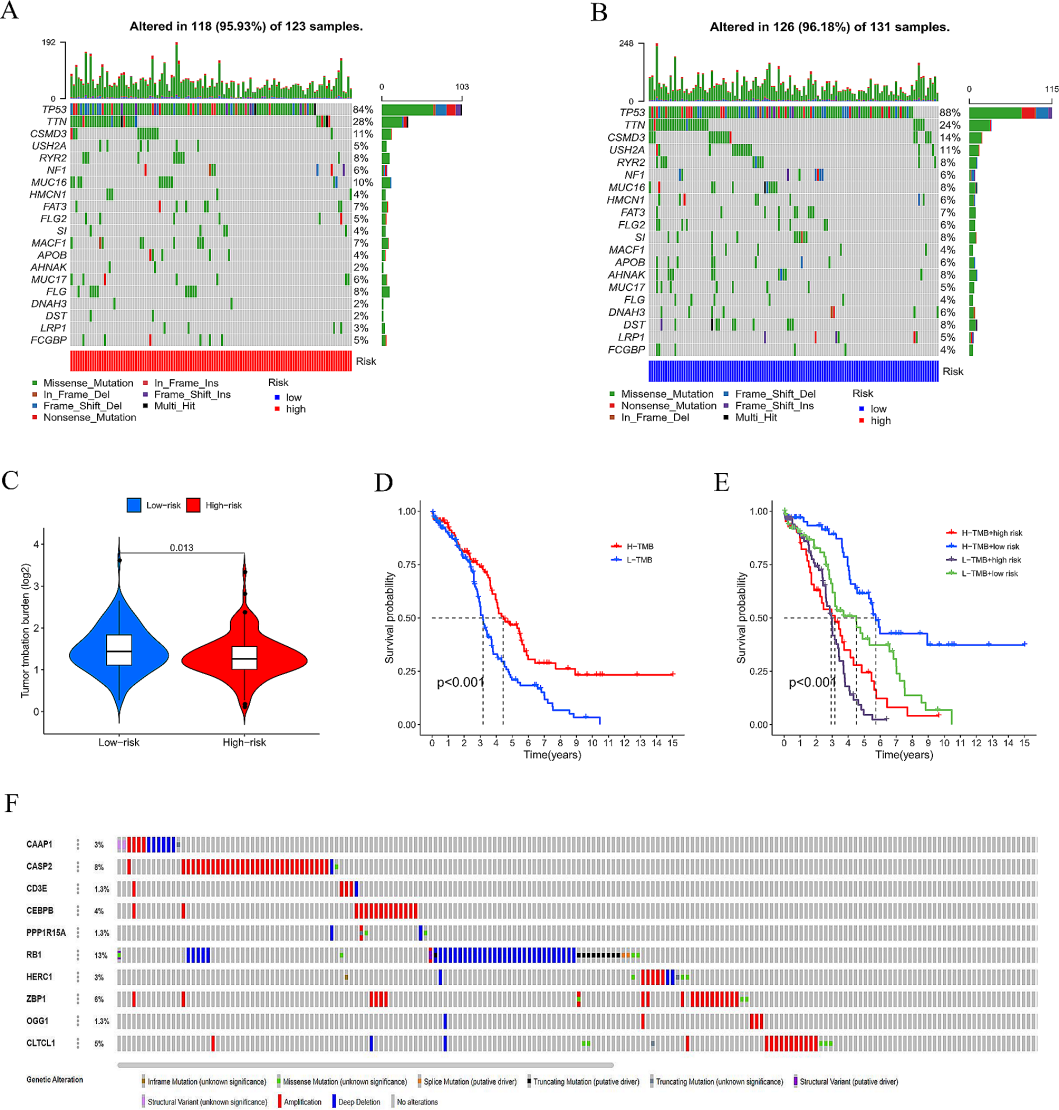
(**A**, **B**) Waterfall maps of the snv data in the high- and low-risk groups. (**C**) Differences of TMB in two groups. (**D**) Survival analysis in high and low TMB groups. (**E**)TMB correlated with risk score groups. (**F**) Mutation rates of ten genes

We downloaded the single nucleotide variants (SNV) data of OC from the TCGA database. The waterfall maps showed the top 20 gene mutations in high-risk and low-risk groups. TP53 was the most common mutation in the two groups (Fig. 9A, B). Higher TMB appeared in the low-risk group compared with the high-risk group (*P* = 0.013) (Fig. 9C), and patients with high TMB gained a longer survival time (Fig. 9D). Figure 9E shows that patients in the low-risk group with high TMB achieved the best OS. The most structural mutations of these ten genes were deep deletion and amplification (Fig. 9F).

### Prediction of potential target drugs

**Fig. 10:**
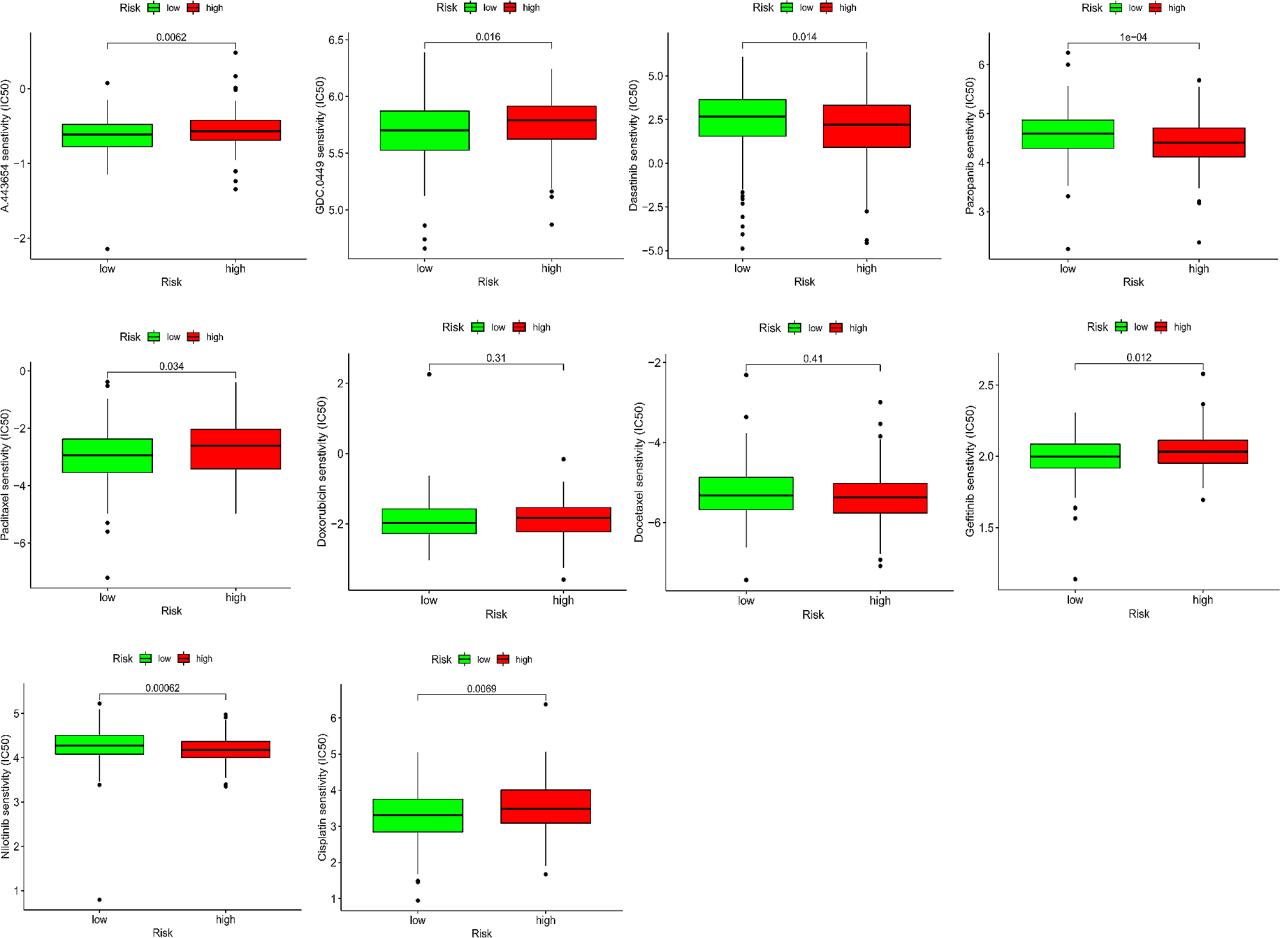
The differences in common chemotherapy drugs between the high- and low-risk groups from the GDSC database

We assessed ten potential target drugs, including A.443,654, GDC.0449, dasatinib, pazopanib, nilotinib, gefitinib, doxorubicin, docetaxel, paclitaxel, and cisplatin. IC_50_ of A.443,654 (*P* = 0.0062), GDC.0449 (*P* = 0.016), paclitaxel (*P* = 0.034), gefitinib (*P* = 0.012), and cisplatin (*P* = 0.0069) in low-risk group were lower than those in high-risk group, indicating that patients with low-risk score would benefit more from these drugs (Fig. 10). And IC_50_ of Dasatinib (*P* = 0.014), Pazopanib (*P* = 0.0001), Docetaxel (*P* = 0.41), and Nilotinib (*P* = 0.00062) in the low-risk group were higher than those in high-risk group, indicating that patients with high-risk score would benefit more from these drugs.

### External validation

**Fig. 11:**
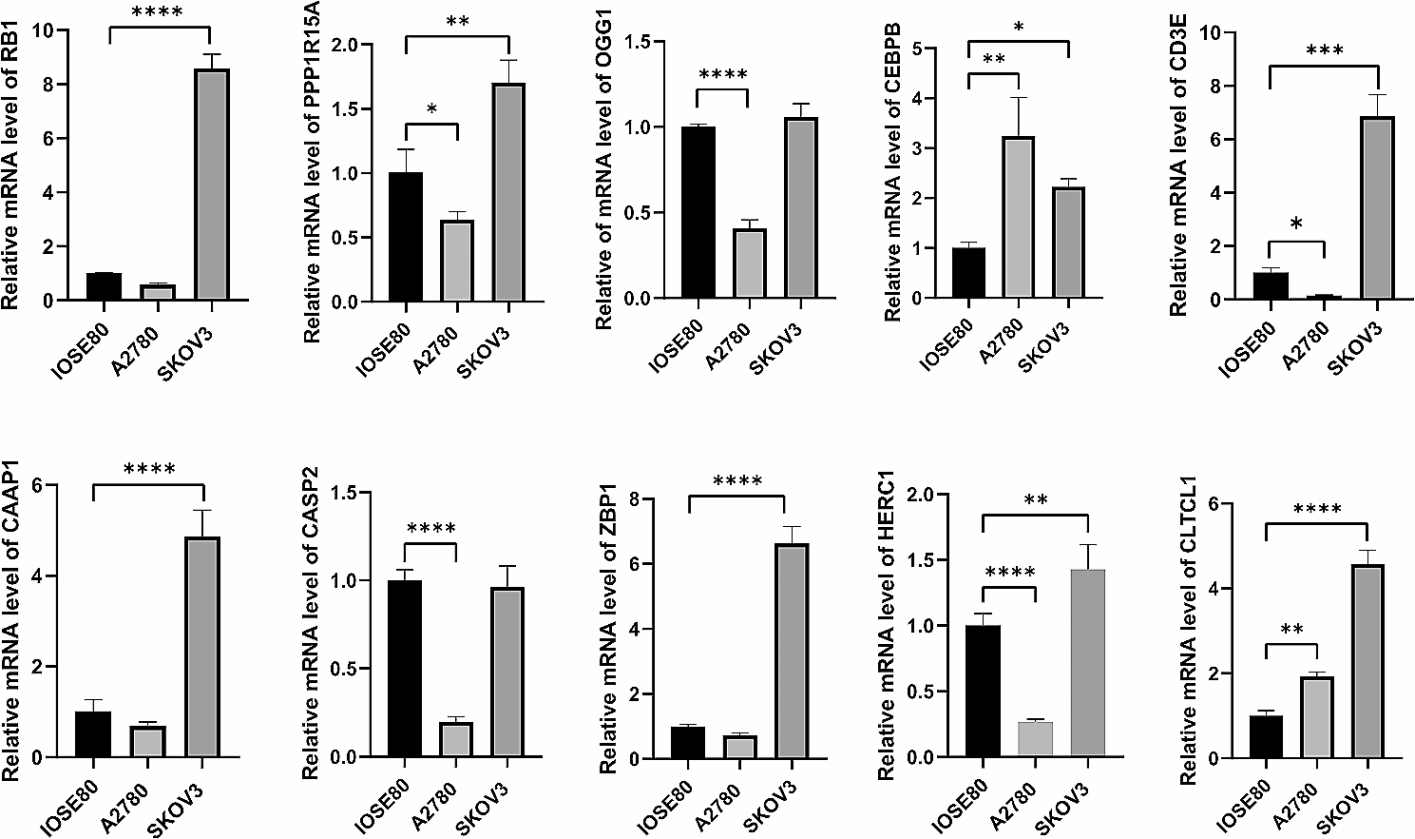
The mRNA expression of ten genes between IOSE80, A2780 and SKOV3

To validate the expression trend of our model genes, we conducted qRT-PCR between normal and ovarian cell lines. We detected higher mRNA levels of CEBPB and CLTCL1 in SKOV3 and A2780 cell lines (*P* < 0.05). Also, the mRNA expression of RB1, PPP1R15A, CD3E, CAAP1, ZBP1 and HERC1 were over-expressed in SKOV3 cells (*P* < 0.05). They showed no difference or underestimated in A2780 cells. We also confirmed a lower expression of OCG1 and CASP2 in A2780 cells (*P* < 0.05). Most of the trend was consistent with the model we created (Fig. 11).

## Discussion

Despite recent advancements in medicine, clinical outcomes of OC patients remain poor due to the advanced stage and chemotherapy resistance [24, 25]. PCD plays a vital role in various aspects in OC, such as tumor development, therapy resistance, and TME [26]. Studies showed autophagy, ferroptosis, and necroptosis could influence the malignant biological properties [27–31]. Autophagy can induce cisplatin resistance in OC patients [32, 33]. Pyroptosis-related genes influence OC tumor immunity [30, 34]. With the in-depth research on PCD’s roles in OC, scholars have realized that PCD-related genes might be helpful prognostic biomarkers and provide novel therapeutic options.

Our study identified a ten-novel PCD-related gene signature that can precisely predict the prognosis of OC patients. Those genes are PPP1R15A, OGG1, HERC1, CASP2, CAAP1, RB1, ZBP1, CD3E, CLTCL1, and CEBPB. Their main functions are listed in Table 1. OC patients were randomly separated into low- and high-risk groups based on the median score. Patients in the low-risk group had better survival outcomes than those in the high-risk group, demonstrating its accurate prediction. The predictive signature had a favorable performance by internal verification and external validation (qRT-PCR). However, further investigations are required to elucidate how these PCD-related genes impact the prognosis of OC patients.

**Table 1:** The biological functions of ten PCD-related genes

Gene	Physiological functions	Inflammation/immune responses	Roles in OC/other cancers
PPP1R15A	integrated stress response regulator [35, 36].	enhances antitumor immunity [37]	influences peritoneal metastasis of OC [38].
OGG1	DNA repairment [39, 40].	promotes DCs activation [41], related to NFκB-dependent inflammatory [42].	arrests cancer cell proliferation [43]. cooperates with TP53 mutations in OC [44]. increase OC susceptibility [45]. induces PARP resistance [46].
HERC1	neurodevelopment, maintain genomic integrity, and cell growth [47].	immune response [47].	regulates breast cancer metastasis [48].
CASP2	participates in apoptosis process and genomic stability [49].	backs up efficient expression of type I interferon [50].	affects colony formation of cancer cells and chemotherapy resistance [51].
CAAP1	inhibit apoptosis pathway [52].	interacts with B or T cells [52].	regulates the apoptosis and autophagy in gastric cancer [53].
RB1	regulates cell cycle progression [54].	enhances immunotherapy sensitivity [54, 55].	predicts the poor prognosis of OC [56, 57].
ZBP1	mediates innate immunity, balance inflammation and cell death [58].	potential target for immune checkpoint blockade inhibitors [59].	regulates cell death in OC via the RIP3/MLKL pathway [60].
CD3E	located on surface of T lymphocytes [61].	adaptive immune response [61] and conduct T cell receptor transmission [62].	a prognostic biomarker for OC patients [63].
CLTCL1	control intracellular traffic, tumorigenesis and cell proliferation [64, 65].	(-)	a favor factor for breast cancer [66].
CEBPB	cell proliferation, differentiation, cell death, and tumorigenesis [67].	monocyte-to-macrophage differentiation [68].	mediate the PARP resistance in OC [69].

It is essential to understand the role model genes play in OC. By GESA analysis, we found our model genes enriched in the ErbB signaling pathway. The ErbB receptor family, also known as the EGF receptor family, includes the epidermal growth factor receptor (EGFR) or ErbB1/Her1, ErbB2/Her2, ErbB3/Her3, and ErbB4/Her4 [70]. Some of our model genes worked via the ErbB signaling pathways. For example, 8-oxo guanine-DNA glycosylase (OGG1) had cross-regulation with the ErbB pathway in thyroid physiopathology [71]. HECT and RLD domain containing E3 ubiquitin protein ligase family member 1 (HERC1) promoted triple-negative breast cancer by regulating the ErbB pathway [72]. RB transcriptional corepressor 1 (RB1) fostered the development of breast cancer by PI3K/AKT signaling [73]. EGFR-targeting molecules could redirect the immune response against tumor cells by tethering effector cells, such as CD3-epsilon (CD3E) T cells, to the surface of cancer cells [74]. The other six genes are uninvestigated in the ErbB pathway. Since the role of the ErbB signaling pathway in OC tumorigenesis was well-established [75–77], we presumed our predictive model could affect OC development through the ErbB signaling pathway. More work is needed to understand their mechanisms in OC.

In recent years, our understanding of the mechanisms of cell death and its consequences on immunity and homeostasis has increased substantially [9]. Our study showed low-risk group presented a higher level of immune infiltration cells, such as dendritic cells (DCs) activated, T cells, CD8 T cells, CD4 memory activated, and T cells follicular helper. DCs are the most widely used cellular vaccination therapy in OC patients [78]. Lymphocytes, such as T cells, CD8 T cells, CD4 memory activated, and T cells follicular helper, are the primary effector cells in cellular immunity. They produce cytokines in immune responses to mediate inflammation and regulate other types of immune cells [79]. In addition, the inflammatory disease, such as endometriosis, is also a contributor to OC. A system review showed that patients with endometriosis have a higher risk of developing ovarian cancer [80, 81]. OC is indeed an immunogenic and inflammatic disease closely tied to those immune cells mentioned above [82]. Although many clinical trials have reported, response rates of various antibodies targeting PD-1 or PD-L1 ranged from 4 to 15% in OC patients [83]. Our study found that the low-risk group presented a higher CD274 (PDL1) and PDCD1 (PD1). Patients with lower risk scores may benefit from PD-1 or PD-L1 inhibitors, such as nivolumab, pembrolizumab, and dostarlimab [34].

Since there are few choices left for the recurrence of OC patients, it is also interesting to consider some potential target drugs. Patients in the low-risk group had higher sensitivity to GDC.0449 and A.443,654. GDC-0449 can inhibit the Hedgehog, a pathway that regulates OC tumorigenesis and chemotherapy resistance [84]. Furthermore, it can improve the antitumor activity of nano-doxorubicin, a common drug for OC patients [85]. Thus, GDC-0449 might be a promising option in OC patients with low-risk scores. A-443,654 is a specific Akt inhibitor. Few studies focus on its role in OC [86]. We found that dasatinib, pazopanib, and nilotinib may benefit OC patients with high-risk scores. Dasatinib was reported as an enhancer to paclitaxel to suppress tumor progression [87]. Pazopanib is now applied in Phase I/II clinical trials for OC patients [88]. Moreover, nilotinib candidates for carboplatin and paclitaxel in OC treatments [89]. However, deeper investigations and clinical trials are still needed to validate the application of these drugs in OC.

In the present study, the PCD-related gene signature is a practical prognostic predictor for OC patients. We demonstrated the association between the risk model and the tumor microenvironment. We also analyzed the potential roles of our model in chemoresistance and immune-related therapy. The PCD-related gene signature could help clinicians stratify high-risk OC patients who need individually additional treatment and intensive follow-up plans. However, our study had some limitations. Firstly, we performed research based on data from the TCGA database and validated it only by PCR. There is still a need for more clinical trials and samples to investigate its potential role in prognosis prediction. Secondly, we should have conducted fundamental experiments to explore the potential mechanisms of PCD-related genes in vivo and in vitro. Thirdly, some potential risk factors, such as gene mutation and therapies that affected OC prognosis, were not brought into our nomogram because of unavailable information in TCGA.

## Conclusions and perspectives

We constructed and validated a predictive signature based on ten PCD-related genes for OC patients (PPP1R15A, OGG1, HERC1, CASP2, CAAP1, RB1, ZBP1, CD3E, CLTCL1, and CEBPB). Our model might help clinicians predict survival outcomes and estimate the therapy response in OC patients. Future work will focus on improving prediction abilities and further testing in experimental research and prospective clinical trials.

### Electronic supplementary material

Below is the link to the electronic supplementary material.

Supplementary Material 1

## Data Availability

The RNA sequencing and clinical data of OC patients can gain from The Cancer Genome Atlas (TCGA) (https://www.cancer.gov/). Information about normal ovary tissue was obtained from GTEx (https://www.gtexportal.org/home/index.html). PCD genes were retrieved from the GeneCards database (https://www.genecards.org). The drugs and their 3D structure can gain from the GDSC database (Home page - Cancerrxgene - Genomics of Drug Sensitivity in Cancer) and PubChem database (https://pubchem.ncbi.nlm.nih.gov).

## Abbreviations

ACD: Accident cell death
CAAP1: Caspase activity and apoptosis inhibitor 1
CASP2: Caspase-2
CD3E: CD3-epsilon
CEBPB: CCAAT/enhancer-binding protein beta
CLTCL1: Clathrin heavy chain like 1
Cmap: Connectivity map
CML: Chronic myeloid leukemia
DCs: Dendritic cells
DEGs: Differentially expressed genes
EGFR: Epidermal growth factor receptor
GDSC: Genomics of Drug Sensitivity in Cancer
GO: Gene Ontology
HERC1: HECT And RLD Domain Containing E3 Ubiquitin Protein Ligase Family Member 1
IC_50_: Half maximal inhibitory concentration
KEGG: Kyoto Encyclopedia of Genes and Genomes
KM: Kaplan-Meier
OC: Ovarian cancer
OGG1: 8-oxoguanine-DNA glycosylase
OS: Overall survival
PPP1R15A: Protein phosphatase 1 regulatory subunit 15 A
PARP: Poly ADP-ribose polymerase
PCD: Programmed cell death
PPI: Protein-protein interaction
Q-PCR: Quantitative Real-Time Polymerase Chain Reaction
RB1: RB transcriptional corepressor 1
ROC: Receiver operation characteristic
SNV: Single nucleotide variants
SsGSEA: Single sample GSEA
TCGA: The Cancer Genome Atlas database
TMB: Tumor mutation burden
TME: Tumor microenvironment
ZBP1: Z-DNA binding protein 1
