# Supplementary material for: A novel TCGA-validated programmed cell-death-related signature of ovarian cancer
Source: BMC Cancer. 2024 Apr 23;24:515. doi: 10.1186/s12885-024-12245-2 (PMC11040780; doi:10.1186/s12885-024-12245-2)
Supplement: Supplementary file 1 — Supplementary Material 1 [file 12885_2024_12245_MOESM1_ESM.docx]

Supplementary Table 1. Clinical pathological parameters of OC patients.

| Clinical | Characteristics | n |
| --- | --- | --- |
| Status | Alive | 282 |
|  | Dead | 305 |
| Futime | <1y | 157 |
|  | ≥1y and <3y | 207 |
|  | ≥3y and <5y | 141 |
|  | ≥5y | 82 |
| Age | Mean (SD) | 59.74 (11.53) |
|  | Median [Min, Max] | 59 [26,89] |
| Grade | G1 | 6 |
|  | G2 | 69 |
|  | G3 | 495 |
|  | Unknow, GX, GB, G4 | 17 |

Supplementary Table 2. Primers of model genes

| Gene |  | Primer sequence |
| --- | --- | --- |
| RB1 | Forward | CTCTCGTCAGGCTTGAGTTTG |
|  | Reverse | GACATCTCATCTAGGTCAACTGC |
| PPP1R15A | Forward | ATGATGGCATGTATGGTGAGC |
|  | Reverse | AACCTTGCAGTGTCCTTATCAG |
| OGG1 | Forward | CACACTGGAGTGGTGTACTAGC |
|  | Reverse | CCAGGGTAACATCTAGCTGGAA |
| CEBPB | Forward | CTTCAGCCCGTACCTGGAG |
|  | Reverse | GGAGAGGAAGTCGTGGTGC |
| CD3E | Forward | TGCTGCTGGTTTACTACTGGA |
|  | Reverse | GGATGGGCTCATAGTCTGGG |
| CAAP1 | Forward | TCTAGCCCAAACGAACCCAAA |
|  | Reverse | CCTCATCTCAAGTTCTAGCAGC |
| CASP2 | Forward | AGCTGTTGTTGAGCGAATTGT |
|  | Reverse | AGCAAGTTGAGGAGTTCCACA |
| ZBP1 | Forward | AACATGCAGCTACAATTCCAGA |
|  | Reverse | AGTCTCGGTTCACATCTTTTGC |
| HERC1 | Forward | TTTGCCCCAACAAGTTTTATGC |
|  | Reverse | GGAGTACAAGCAGTCGTTTTCT |
| CLTCL1 | Forward | GGTCACGATCATTGACATGAGT |
|  | Reverse | GCTATCACCTTAGAGGCTGGAT |
| GAPDH | Forward | TGTGGGCATCAATGGATTTGG |
|  | Reverse | ACACCATGTATTCCGGGTCAAT |
